# Advances in Computational Intelligence-Based Methods of Structure and Function Prediction of Proteins

**DOI:** 10.3390/biom14091083

**Published:** 2024-08-29

**Authors:** Jian Zhang, Jingjing Qian

**Affiliations:** School of Computer and Information Technology, Xinyang Normal University, Xinyang 464000, China; 2023211107@xynu.edu.cn

Proteins serve as the building blocks of life and play essential roles in almost every cellular process. Protein sequence refers to the linear arrangement of amino acids, which are connected by peptide bonds. The protein sequence provides the blueprint for the corresponding secondary and three-dimensional (3D) structures [1,2]. As is well known, the protein’s sequence determines its structure. However, given various environmental conditions, post-translational modifications, and interactions with other ligands, the same protein sequence may form different 3D structures [3]. Experimental structure determination and function analysis techniques are usually time-consuming and labor-intensive [4], which can benefit from computational intelligence-based models and approaches [5]. Computation-based methods have significantly contributed to protein structure prediction by offering a powerful suite of tools and algorithms that can accurately model and simulate the 3D conformations of proteins. These methods are invaluable in bridging the gap between the protein’s primary sequence and the complex, functional architecture of a protein’s tertiary shape. CASP (Critical Assessment of Structure Prediction) is a community that invites participants to provide their models to identify unknown protein structures [6]. This competition is held every two years. The latest CASP15 in 2023 attracted about 100 groups of researchers and received over 53,000 models on 127 modeling targets [7]. These methods of prediction of protein structures include: (i) template-based modeling from known protein structures; (ii) ab initio modeling via sophisticated energy functions or conformational search techniques; and (iii) artificial intelligence-aided methods that employ machine learning or deep learning algorithms. Table 1 collects the representative predictors for each category mentioned above. We selected three popular predictors for each type of protein structure prediction method. The citations are used as a direct way to quantify the impact of these resources within the community [8]. The citation counts were collected from Google Scholar (https://scholar.google.com/) on 16 August 2024.

Specifically, template-based modeling methods attract the most attention among bioscientists. These methods require high-resolution, accurate protein structure/template databases [9]. This type of method tries to find the most similar templates to model the structure of the target, unknown protein. The template-based modeling methods are regarded as the most successful and widely used approaches [9,10]. However, they may fail when proper orphan proteins or templates are not available. Then, it usually relies on pre-designed physical principles and statistical potentials to predict the structure only from the protein primary sequence. This type of method belongs to ab initio modeling. Recent years have witnessed the rapid development of artificial intelligence (AI) techniques. With the development of computing power, especially the rise of GPU computing, GPU-accelerated methods make it possible for both efficient and accurate protein structure modeling. The AI-aided modeling methods infer protein structures via machine learning and deep learning algorithms [11]. Currently, AI-driven methods like AlphaFold [12] are leading the research. In the near future, we can expect more accurate and efficient approaches for predicting protein structures.

**Table 1:** Representative predictors of template-based, ab initio, and AI-aided modeling methods of protein structure. These research papers are sorted by the number of citations (scientific impact) in descending order.

Types of Methods	Representative Methods	Year	Citations	Reference
Template-Based Modeling	MODELLER	2016	10,180	[13]
	I-TASSER	2015	6060	[14]
	Sparks-X	2011	375	[15]
Ab Initio Modeling	QUARK	2012	1123	[16]
	RaptorX-Property	2016	574	[17]
	QMCPACK	2018	293	[18]
AI-Aided Modeling	AlphaFold	2021	25,578	[12]
	RaptorX_Contact	2019	417	[19]
	trRosetta	2021	404	[20]

In cells, all proteins have specific biological activities or functions, including structural supports and movements, enzymatic activities, and interactions with other ligands [21]. Protein functions depend on the corresponding 3D structures. For instance, Zhang et al. pointed out that the ligands tend to be located in the relatively small cavities of the protein surface [22]. If enzymes have buried active sites, substrates need to pass through the body of the protein in order to bind these sites [23]. According to recent studies [24,25,26,27], the approaches that predict protein function from structure include structure alignment, molecular docking, and AI-aided prediction. Researchers use Gene Ontology (GO) to detail the functions of a protein. The GO information includes biological process ontology, molecular function ontology, and cellular component ontology [28,29]. Figure 1 summarizes the protein relationships among sequence, structure, and function.

This Special Issue consists of eight original research articles and one review of this topic. The research articles provide the readers with the latest developments in protein folding, secondary structure prediction, the HtrA protease family, SARS-CoV-2 spike variant complexes, heme distortion, Trp305, enzyme substrate promiscuity, and the hepatitis C virus genome. The review summarizes and compares existing network-based methods for predicting drug-disease associations.

Azulay et al. propose an interesting idea that compares and analyzes the similarity between proteins and origami [30]. Although the two things are not identical, they share some equivalences. Protein folding is driven by the physical arrangement of the residue chain and chemical forces. The corresponding crease patterns, like mountains or valleys, also appear in origami. The origami crease patterns and folding inspire scientists to explore protein folding constraints, properties of folded mediums, and folding energy. Besides that, the authors also discuss several unique mechanical properties, which have high stiffness-to-mass ratios and excellent abilities to withstand high forces. The capability of translating origami models to protein structures promises the visual design of de novo proteins and nanomaterials with the desired properties.

In [31], Guo et al. propose a novel method called CondGCNN to predict protein secondary structure. CondGCNN combines a conditionally parameterized convolutional network and a gated convolutional neural network. Particularly, the encoder layer of CondGCNN utilizes both the long short-term memory network and CondGCNN to compute protein sequential features. The results on benchmark testing datasets and a set of CASP datasets prove the good performance of their method.

Merski et al. investigate the structure-repeating module in the HtrA protease family [32]. They use a self-homology detection method based on a modified version of DOTTER [33] to analyze these protein sequences. As a result, they find a 26-residue segment/pattern, which forms an anti-parallel *β*-barrel structure. By using MUSCLE [34], the authors find that 13 out of 26 positions are evolutionary conserved. This repeating architecture has gone unnoticed, although these structures have been publicly available for two decades.

Verkhivker et al. perform a computational analysis of SARS-CoV-2 receptor-binding domain Omicron complexes with several ultra-potent antibodies [35]. They find that the dominant binding energy hotspots and allosteric centers of long-range interactions in the Omicron complexes share the same set of residues: Y449, Y453, L455, F486, Y489, and F490. These residues are conserved and hydrophobic with a low probability of mutation and are important for the receptor-binding domain and binding with the host receptor. If mutations occur in these residues, they will severely impair binding with the antibodies.

Compared to the isolated structure, heme in the host protein exhibits various degrees of distortion [36]. Moreover, the doming distortions in the oxygenated and deoxygenated states differ between hemoglobin and myoglobin [37]. In other words, the protein environment affects the heme molecular structure and controls the chemical properties of heme [22]. Kondo et al. construct a convolutional neural network to predict the distortion of heme from the 3D structure of the heme-binding pocket [38]. They examine the correlations between the shape of the cavity and the molecular structure of heme and obtain high correlation coefficients for saddling, ruffling, doming, and waving distortions.

The death-associated protein kinase (DAPK) family regulates important biological functions in human cells [39]. Among them, DAPK1 is the largest protein in its family and functions as a drug target in some diseases, such as cancer and Alzheimer’s disease [40]. The research of Zhu et al. investigates the molecular mechanism of Trp305 (W305Y and W305D) in modulating DAPK1 activity [41]. They conclude that the W305D mutation enhanced the anti-correlated motions between DAPK1 and calcium/calmodulin, and the latter can interact with the W305Y DAPK1 mutant.

EP-Pred, a machine learning tool proposed by Xiang et al., is designed for identifying enzyme substrate promiscuity [42]. It adopts Possum [43] and iFeature [44] to extract evolutionary information and physicochemical properties, respectively. After feature selection, the authors use SVM, KNN, and RidgeClassifier to construct an ensemble binary predictor. They use a hidden Markov approach to select promiscuous esterases from the Lipase Engineering Database. The EP-Pred confirms the validity of the selection and correctly recognizes all ten proteins.

Hepatitis C virus (HCV) infection is a major cause of liver failure and hepatocellular carcinoma worldwide [45]. In [46], Zhuang et al. aim to investigate how the stem-loop 1 and the 9th nucleotide of HCV affect the conformation and dynamics of the Ago2: miRNA: target RNA complex. They perform molecular dynamics simulations on the Ago2-miRNA complex and design a model wherein the Ago2 protein can adopt a more constrained conformation to protect the target RNA from dissociation. They find the mechanism of the Ago2-miRNA complex’s protective effect on the HCV genome. This conclusion promises to offer guidance for the development of anti-HCV strategies.

The review by Kim et al. collects and compares the recently released network-based methods for drug-disease association prediction [47]. It categorizes these methods into three groups: graph mining, matrix factorization/matrix completion, and deep learning. The authors adopt two uniform datasets as the benchmark testing datasets. The two datasets are used to predict associations on the drug side and the disease side, respectively. Kim et al. compare the performance of the selected 11 predictors. Based on the experimental results, they find that the methods with graph mining and matrix factorization/matrix completion show better results than those with deep learning. Moreover, the current methods have higher accuracy on the drug side than on the disease side.

We hope that the readers will enjoy reading this Special Issue of Biomolecules and that the research articles on protein folding, secondary structure prediction, HtrA protease family, SARS-CoV-2 spike variant complexes, heme distortion, Trp305, enzyme substrate promiscuity, and hepatitis C virus genome, and the review on drug-disease associations will help advance the field and provide new ideas to researchers.

## Figures and Tables

**Figure 1:**
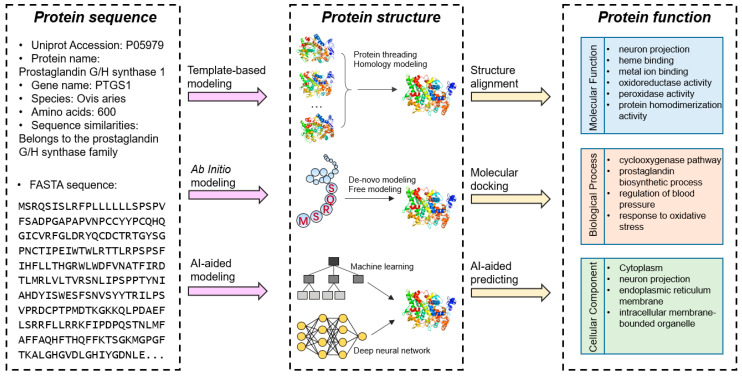
A schematic view of the protein sequence-structure-function relationships. Generally, protein sequence determines structure, and structure determines function.
